# Reptilian-transcriptome v1.0, a glimpse in the brain transcriptome of five divergent Sauropsida lineages and the phylogenetic position of turtles

**DOI:** 10.1186/2041-9139-2-19

**Published:** 2011-09-26

**Authors:** Athanasia C Tzika, Raphaël Helaers, Gerrit Schramm, Michel C Milinkovitch

**Affiliations:** 1 Laboratory of Artificial & Natural Evolution (LANE), Dept. of Genetics & Evolution, University of Geneva, Sciences III, 30, Quai Ernest-Ansermet, 1211 Genève 4, Switzerland; 2 Laboratory of Human Molecular Genetics (GEHU), de Duve Institute, Université catholique de Louvain, Avenue Hippocrate 75-50, B-1200 Brussels, Belgium; 3 Roche Diagnostics GmbH, Global Systems Support, 2 Nonnenwald, 82377 Penzberg, Germany

## Abstract

**Background:**

Reptiles are largely under-represented in comparative genomics despite the fact that they are substantially more diverse in many respects than mammals. Given the high divergence of reptiles from classical model species, next-generation sequencing of their transcriptomes is an approach of choice for gene identification and annotation.

**Results:**

Here, we use 454 technology to sequence the brain transcriptome of four divergent reptilian and one reference avian species: the Nile crocodile, the corn snake, the bearded dragon, the red-eared turtle, and the chicken. Using an in-house pipeline for recursive similarity searches of >3,000,000 reads against multiple databases from 7 reference vertebrates, we compile a reptilian comparative transcriptomics dataset, with homology assignment for 20,000 to 31,000 transcripts per species and a cumulated non-redundant sequence length of 248.6 Mbases. Our approach identifies the majority (87%) of chicken brain transcripts and about 50% of *de novo *assembled reptilian transcripts. In addition to 57,502 microsatellite loci, we identify thousands of SNP and indel polymorphisms for population genetic and linkage analyses. We also build very large multiple alignments for Sauropsida and mammals (two million residues per species) and perform extensive phylogenetic analyses suggesting that turtles are not basal living reptiles but are rather associated with Archosaurians, hence, potentially answering a long-standing question in the phylogeny of Amniotes.

**Conclusions:**

The reptilian transcriptome (freely available at http://www.reptilian-transcriptomes.org) should prove a useful new resource as reptiles are becoming important new models for comparative genomics, ecology, and evolutionary developmental genetics.

## Background

The field of comparative genomics is constantly enriched by the addition of newly sequenced genomes: by the end of 2010, about 1,300 bacterial and 150 eukaryotic genomes had been sequenced http://www.genomesonline.org with various degrees of precision and coverage. In particular, there is a great interest in mammalian genomes, given their proximity to humans and, hence, their potential power for generating biomedically-relevant data. Identification of conserved elements has been a central focus of comparative analyses and the driving force behind initiatives such as the *'Multiple Mammalian Genomes for Comparative Annotation' *project http://www.genome.gov/25521745, initially including 24 mammalian species. The recent development of next-generation sequencing technologies [1-3] allows the comparative genomics community to contemplate the possibility of incorporating high-coverage full genome sequences from many non-classical model organisms for a better understanding of how biological diversity and complexity evolved. For example, the *'Evolution of the Human Proteome' *initiative aims at sequencing the genome of nine additional chordate species to complete the coverage of major lineages of the chordate phylogeny and uncover the genomic changes that correlate with key morphological and physiological transitions http://www.genome.gov/25521740.

Among under-represented groups, in terms of genome sequence data, are the major lineages of Sauropsida, which diverged 200 to 280 million years ago: Testudines (turtles), Lepidosauria (the tuatara, lizards, and snakes) and Archosauria (crocodiles and birds). Even if we exclude the 10,000 extant species of birds, Sauropsida still includes over 8,000 species (compared to 5,400 species of mammals) that display a remarkable range of life histories, sex-determining systems, reproductive modes, physiologies, and body plans [4]. For example, in squamates, limb reduction has evolved independently at least 25 times [5], and viviparity at least 100 times [6] (*versus *less than 15 times each in bony fishes, cartilaginous fishes, and amphibians, once in mammals, and never in birds); shifts between genetic and temperature-dependent sex determination have occurred multiple times as well [7]; and some lizards even exhibit ovulation of tiny eggs and placental nutrition of embryos [8]. Hence, comparative genomic analyses incorporating reptilian genomes promise to uncover evolutionary novelties more diverse in many respects than those revealed by genomic comparisons among mammals. Furthermore, non-avian reptilian genomes would greatly improve the comparison between mammals and birds by incorporating major missing nodes between these two lineages [9,10]. Thus far, only the genome of the green anole lizard (*Anolis carolinensis*) and a handful of birds (the chicken, *Gallus gallus*; the zebra finch, *Taeniopygia guttata*; the duck, *Anas platyrhynchos*; and the turkey, *Meleagris gallopavo*) have been fully sequenced. Model reptilian species, whose genome should be sequenced in priority, need to be chosen pragmatically [11,12] by incorporating criteria such as phylogenetic position, nature of the ancestral/derived states of key morphological/physiological characters, level of diversity within the corresponding higher taxon, ease with which the species can be handled, housed and bred, and protection status.

Even if next-generation methods make the sequencing of a complex genome possible in a matter of weeks, such a project remains very costly and requires much additional time for assembly and annotation. For species that are considerably divergent from existing high-quality genomes, gene identification and annotation greatly benefits from transcriptome data. Again, next-generation sequencing will probably become the method of choice for generating high-quality transcriptome data and supplant other methods such as serial analysis of gene expression (SAGE), sequencing of expressed sequence tags (ESTs), substractive hybridization, differential display, and even microarrays (at least for non-model species). Indeed, next-generation sequencing of transcriptomes has recently proven to be highly valuable for producing functional genome sequences, as well as gene polymorphism and expression data [13-17]. In addition, software has been developed for handling the massive amount of sequence data and for *de novo *assembling of contigs without the need of reference genomes [18,19].

Besides large-scale EST libraries available for several organs of the anole lizard (including a brain library, dbEST library #23338, yet to be analyzed), reptilian transcriptomes so far are quite limited: a few snake venom-gland partial transcriptomes (each consisting of 600 to 1,000 ESTs generally clustering into about 300 unique sequences [20-22]), a heart transcriptome of the Burmese python consisting of about 2,800 mRNAs [23], 3,064 assembled unique sequences of *Alligator missipiensis *analyzed for their GC-content [24], and 833 assembled unique sequences available for the red-eared slider turtle, with a few related to brain development [25,26]. A notable very recent exception is a garter snake large-scale multi-individual and multi-organ transcriptome [27], which identified about 13,000 snake genes on the basis of homology assignment with other vertebrates, as well as thousands of transcripts of unidentified protein-coding genes.

Here, we used 454 technology for sequencing brain transcriptomes in four reptilian and one avian species: (i) the Nile crocodile (*Crocodylus niloticus*), whose development has recently been described [28], (ii) the oviparous Corn snake (*Elaphe guttata*), as a better alternative (in the Evo-Devo context [11,12]) to the viviparous common garter snake (*Thamnophis sirtalis*), (iii) the Bearded dragon (*Pogona vitticeps*), a lizard of the Agamidae family that diverged approximately 150 mya from the Iguanidae [29] to which *Anolis *belongs, (iv) the red-eared turtle (*Trachemys scripta*), and (v) the chicken (*G. gallus*) as a reference for the performed analyses. We chose to focus on the brain for one primary reason: it exhibits one of the most complex (*that is*, diverse) transcriptomes of all organs in vertebrates [30,31]; hence, it is a tissue of choice for sequencing a maximum number of transcripts while reducing the need for normalization. Note also that reptilian species have been incorporated in comparative analyses of the vertebrate brain [32] aimed at understanding the evolution of the sensory and cognitive novelties associated with the vertebrate central nervous system [33,34], a topic beyond the scope of the present paper.

We generated over 3,000,000 reads which were fed into an automated and publicly-available pipeline, '*LANE runner*', that performs iterative BLAST searches and consensus assemblies. A total of 20 to over 31 thousand genes were identified per species, including transcripts that might be lineage specific. This new reptilian comparative transcriptomics dataset (available at http://www.reptilian-transcriptomes.org) should prove a useful resource as reptiles are becoming important new models for comparative genomics (*for example*, [35]), ecology (*for example*, [4]), and evolutionary developmental genetics (*for example*, [36-38]). We also identify thousands of both microsatellite loci and SNPs which can be used in quantitative and population genetic analyses. Finally, we built the longest (2,012,759 amino acids (aa)) reptilian multiple alignment of homologous sequences to date (found in all five lineages of Sauropsida, three mammals, and two outgroup taxa) and performed extensive phylogenetic analyses for investigating the long-standing question of the turtle lineage position within the phylogeny of Amniotes. Although phylogenetic results must be taken with caution, as sequencing errors in low coverage transcriptomes could generate artifacts during phylogeny inference, maximum likelihood analyses of a large dataset (about 250 thousand characters per species) void of paralogs hint at archosaurian affinities of Testudines.

## Methods

### cDNA library construction and sequencing

The complete brains of a crocodile (*C. niloticus*), a corn snake (*E. guttata*), a bearded dragon lizard (*P. vitticeps*), a red-eared slider turtle (*T. scripta*), and a chicken (*G. gallus*) were each placed in the appropriate amount of RNAlater (QIAGEN, Germantown, MD, USA) and homogenized using MixerMill (Retsch Haan, Germany). mRNA extraction and first strand cDNA synthesis were performed using the 'μMACs One-Step cDNA synthesis kit' (Miltenyi, Biotech Bergisch Gladbach, Germany), according to the manufacture's protocol. The eluted cDNA/mRNA hybrids were directly used for the second strand cDNA synthesis, in the presence of *Escherichia coli *ribonuclease H and *E. coli *DNA polymerase I (Fermentas, Canada). After a 2-hour incubation at 16°C, the samples were treated with *E. coli *DNA ligase (Invitrogen, Carlsbad, CA, USA) and T4 DNA polymerase (Fermentas) for filling-in nicks and blunting ends, respectively. Products were phenol/chloroform extracted before ligation of unphosphorylated double-stranded adaptors (OligoI: 5' - AAGCAGTGGTATCAACGCAGAGTAC - 3' and OligoII: 5' - GTACTCTGCGTTGATACCACTGCTT - 3'). The adaptor sequence corresponds to the 'CAP' primers (Clontech, Mountain View, CA, USA) to take advantage of the PCR suppression effect to preferentially amplify longer molecules and enrich for full-length transcripts [39]. Prior to amplification of the ligation product, a fill-in step was performed to remove the 5'-end nick between OligoII and the cDNA: a mix of the ds-cDNA+adaptors, dNTPs, LongExpand polymerase and PCR buffer 1 (Roche, Basel, Switzerland) was incubated at 68°C for 5 minutes. OligoI was then added as a primer for cDNA amplification: denaturation at 93°C for 2 minutes followed by 10 cycles of 93°C for 10 seconds, 60°C for 30 seconds, 68°C for 7 minutes, and 20 cycles of 93°C for 15 seconds, 60°C for 30 seconds, 68°C for 7 minutes + 20 seconds/cycle. The cDNA library construction and sequencing was performed as in [1].

### Contig initial assembly

All sequence and quality files were merged (with *'A.F.7 Merge files' *v1.3) separately for each species. Removal of adaptor sequences is built in our in-house software, 'LANE runner' (see below): adaptor sequences identified at the beginning or end of a read were removed along with the corresponding quality values. In rare cases, when the adaptor was identified within the sequence rather than at its extremities, the adaptor sequence was removed and the read (and associated quality values) was split into the two corresponding parts. Near-exact matches to adaptors and PolyA stretches were removed, and the remaining sequences were assembled into contigs, using *SeqMan NGen v2.0 *(DNASTAR). Default parameters for *de novo *assembly of '454' reads were used, except for two settings: the '*Match Spacing*' was set to 50 (instead of 10) and the '*Min Match Percentage*' was set to 80 (instead of 85). The first parameter represents the length of the window of a sequence read where at least one mer tag (*that is*, a unique subsequence) will be searched for, and the second is the minimum percentage of identity between two reads for every alignment of 50 bases before extension of alignment is attempted. Unassembled reads (singletons) were trimmed based on sequence quality.

### Homology assignment using BLAST

Zero to four reads per species assembly were identified as bacterial contaminants and were removed. The contigs and singletons were first aligned (BLAST) against the following databases: (i) *'Ensembl ncRNA v. 56'*, containing known non-coding RNA molecules, such as ribosomal, transfer, or micro RNAs, and (ii) '*mtDNA*', comprised of the mitochondrial genome available at NCBI for each of the five species (accession numbers: AJ810452, AM236349, AP003322, NC_006922, NC_011573). We restricted the searches against the Ensembl non-coding database of two mammalian species (*Homo sapiens*, *Mus musculus)*, one lizard (*A. carolinensis)*, two birds (*G. gallus*, *Taeniopygia gutatta*), one amphibian (*Xenopus tropicalis*), and one fish (*Danio rerio*). All searches were performed with our in-house JAVA application, *LANE runner*, (available at http://www.reptilian-transcriptomes.org) that provides a user-friendly interface for: (i) defining distinct BLAST settings for each searched database, (ii) submitting input sequences to a *'wwwblast' *local server, and (iii) parsing and summarizing all results in an XLS file. For the non-coding and mtDNA databases, the *'blastn' *settings were: *e*-value threshold = 10, gap opening and extension penalties set to 5 and 1, respectively, word size = 11, mismatch cost = 1, and match award = 2. Input sequences were masked with the 'low complexity' filter incorporated in BLAST. Hits with a match length <50 bp and/or an identity <50% were rejected.

**Figure 1:**
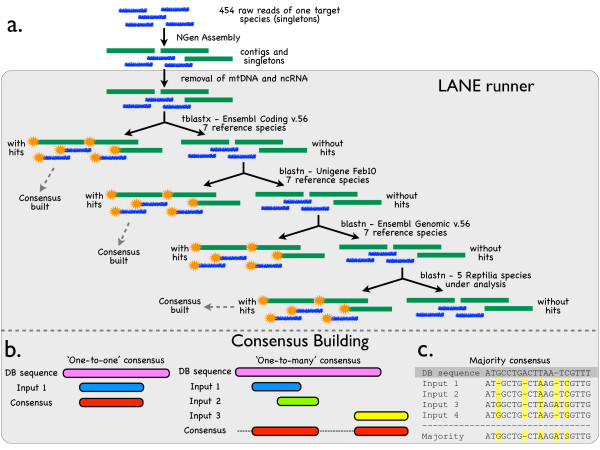
**Our sequence data analysis pipeline including (a) contig assembly and homology assignment and (b and c) consensus building**.

All input sequences (contigs and singletons) with a hit against the '*mtDNA*' database were reassembled (using *NGen v.2*) with the mitochondrial genome sequence of the corresponding species used as a reference. The sequences (contigs and singletons) not included in the mtDNA assembly, nor with a hit against the non-coding RNA database, were subjected to four additional rounds of BLAST comparisons (Figure 1). At each round, the input sequences with a hit were removed and used to built a consensus (see below), whereas the remaining ones were passed to the next round. The first round of BLAST was against the *'Ensembl Coding v56' *database, which contains all known transcripts (that can be mapped to species-specific entries in public protein databases), novel transcripts (*for example*, genes predicted on the basis of evidence from closely related species), and pseudogenes, along with their 5' and 3' untranslated regions (UTRs). The *'tblastx' *settings were: *e-*value threshold = 10, gap opening and extension penalties set to 11 and 1, respectively, word size = 3, and *'BLOSSUM45' *protein substitution matrix. Input sequences were masked with the 'low complexity' filter. Hits with a match length <30 aa (*that is*, 90 bp) and/or an identity <50% were rejected. The second round of BLAST was against the '*Unigene February 2010*' database containing the longest high-quality sequence from each Unigene cluster. *'Blastn' *settings were as for the '*mtDNA*' comparisons except that word size was set to 9. For the third round, sequences with no hit against any of the previous databases were aligned (BLAST) against the '*Ensembl Genomic v56*' database containing the full genome of each reference species (*'blastn' *settings as for the Unigene analysis). Finally, the remaining sequences of each species were compared to the consensus sequences resulting from the previous BLAST rounds in the four other species (*'blastn' *criteria as for the Unigene analysis). Input sequences still with no hit were masked using the *RepeatMasker *Web Server (version open-3.2.9; [40]) with the 'cross_match' search engine and 'Chicken' as DNA source. All possible ORFs in species-specific transcripts longer than 1,000 bp were identified using *ORF Finder *[41], and the longest deduced amino-acid sequence was aligned (BLAST) against the *'nr' *NCBI database.

### Building and analysis of consensus sequences

Consensus sequences were built using *LANE runner*http://www.reptilian-transcriptomes.org based on the BLAST results. As one query sequence can hit several sequences in one database, only the best match was chosen on the basis of the following successive criteria: smallest *e*-value, greatest match length, and highest percent of identity. If all values were equal between different hits, the input sequence was attributed to the first best result encountered to avoid redundancy. When the database consisted of nucleotide sequences, the orientation of each input sequence (forward or reverse complement) yielding the best alignment score against the database sequence was identified with *JAligner *[42], whereas, for the protein databases, the input sequences were translated using the frame indicated by the best BLAST match. The query sequences were aligned against the database sequences using *MUSCLE*[43]* via *the EBI server. 'LANE runner' automatically retrieved alignments and computed a majority consensus (Figure 1c). When the alignment was performed with protein sequences (*that is*, on the basis of *'tblastx' *results), it was first back-translated into the original nucleotide sequence before building the consensus. The database (reference) sequence is only used for anchoring input sequences and not for computing the consensus itself, hence, the portions of the reference sequence that do not match any input sequence are replaced by gaps (Figure 1b). We cannot exclude the possibility that different sections of paralogous genes are joined during this procedure. When only one input sequence corresponded to one database reference sequence, no alignment was made and the 'one-to-one consesus' comprised only of the input sequence. Consensus sequences were named after the corresponding database reference sequence. '*LANE runner*' also computes gap percentages in three user-defined regions of each consensus: here, the first (5') 30%, the middle 40%, and the final 93') 30% (Additional file 1, Table S1).

### Identification of SSR loci and SNPs

The assembled contigs and singletons were processed with *MISA *[44], for the identification of perfect and compound microsatellites. Monomers were taken into account when repeated more than 10 times and di- to hexa-nucleotides more than 5 times. Two or more perfect microsatellites separated by less than 50 bp were considered as a single compound microsatellite. High-confidence SNPs in nuclear DNA contigs were detected with *NGen v2*, using a minimum sampling depth of three for the minor allele (to discriminate sequencing errors from genuine polymorphisms [45]). Note that, as we used a single individual brain for each species, these polymorphisms correspond to heterozygous sites.

### Phylogeny inference

We identified 4,689 genes for which at least partial aa sequences were found in both our *C. niloticus *and *T. scripta *transcriptomes. The homologs (when available) were retrieved from the transcriptomes of the three other species (*E. guttata*, *P. vitticeps*, and *G. gallus*) as well as from Ensembl v56 for *D. rerio*, *X. tropicalis*, *A. carolinensis*, *G. gallus*, *T. guttata*, *M. musculus*, *H. sapiens*, and *O. anatinus*. For each protein, all aa sequences were aligned using the *MUSCLE *server at EBI ([43]; http://www.ebi.ac.uk/Tools/webservices/services/muscle). When no Ensembl *Gallus *sequence was available, our *Gallus *sequence was used. New datasets were built with *trimAl*[46] by (i) removing all gaps or (ii) removing the positions that define the maximum drop in the sorted distribution of gap scores. Additional protein sequence datasets were generated by combining sequences from different species into hybrid sequences (Additional file 1, Figure S4). We performed analyses with various combinations of hybrids: the two birds, the three squamates, and the two non-amniote outgroup species. Different species priorities were also tested, *for example*, using the *Gallus *sequence only when the *Taeniopygia *sequence is not available, or *vice versa*.

To remove portions of the alignment where sequences might be paralogous, we selected in *MANTiS*[47] the protein sequences in our reptilian transcriptome dataset for which none of the corresponding gene in *D. rerio*, *X. tropicalis*, *A. carolinensis*, *G. gallus*, *T. guttata, M. musculus*, *H. sapiens*, and *O. anatinus *exhibits paralogs.

All protein sequence alignments were phylogenetically analyzed (with *D. rerio *and *X. tropicalis *as outgroup taxa) under the WAG or GTR maximum likelihood (ML) aa substitution models and rate heterogeneity, with *RaxML v7.2.6 *[48] using 100 bootstrap replicates, and with *MetaPIGAv2.1 *[49] a software available at http://www.metapiga.org and implementing the metapopulation genetic algorithm [50] together with complex substitution models, discrete gamma rate heterogeneity, and the possibility to partition data. For *MetaPIGA* analyses, we used probability consensus pruning among four populations of four individuals each, and selected the best-fitting ML nucleotide substitution model (GTR and gamma-distributed rate variation across sites) on the basis of the Akaike Information Criterion implemented in *MetaPIGA*. To generate an estimate of the posterior probability distribution of possible trees, we performed replicated metaGA searches and stopped when a series of mean relative error values [49] among 15 consecutive consensus trees remained below 2% (with a minimum of 100 replicates).

## Results and discussion

### Transcriptome sequencing and contig initial assembly

Using a GS FLX genome analyser (454/Roche), we sequenced amplified double-strand (ds) cDNA on two plates for *C. niloticus*, *P. vitticeps *and *T. scripta*, and one plate and a half for *E. guttata *and *G. gallus*. The number of raw reads per species ranged from 524 thousands to 884 thousands (Table 1), with an average read length of 164 to 207, and 92% to 98% of the reads passed the quality filters (*that is*, removal of low-quality, polyA stretches, adaptors or artifactual sequences).

**Table 1:** Statistics of the 454 sequencing: number of plates, raw reads, discarded reads, and average read length

	*Gallus*	*Crocodylus *	*Elaphe*	*Pogona*	*Trachemys*	All
Plates	1.5	2	1.5	2	2	9
Raw reads	558,538	523,785	554,054	884,080	613,632	3,134,089
Discarded	13,484 (2.4%)	42,284 (8.1%)	9,139 (1.7%)	15,591 (1.8%)	30,320 (4.9%)	110,818 (3. 5%)
Av read length	191	181	207	191	164	187

Using *NGen *(DNAStar), we assembled between 25,819 and 52,348 contigs (depending on the species, see Table 2 for details) for a cumulated contig length of 10.1 to 21.6 Megabases (Mb) per species, whereas 168,075 to 263,428 reads remained unassembled (='singletons'). When considering both contigs and singletons, the cumulated total length of unique sequences per species amounted 37.6 Mb (*Trachemys*) to 69.0 Mb (*Pogona*). The average contig length was 360 to 424 bp, but a substantial number of larger contigs was also observed: *for example*, 10,709 *Pogona *contigs were >500 bp, and 2,792 were >1 kb. The longest nuclear and mitochondrial contigs were assembled for *Pogona *(6,063 bp) and *Crocodylus *(7,513 bp), respectively. On average, each contig contained 9.5 to 13.2 reads and the average sequence depth was 2.9 to 3.7 (Table 2). Distributions of contig size, number of reads per contig, and contig lengths are shown in Additional file 1 (Figure S1).

**Table 2:** Statistics of NGen assembly (*mt*: mitochondrial DNA)

	*Gallus*	*Crocodylus*	*Elaphe*	*Pogona*	*Trachemys*
Contigs generated	39,723	36,088	25,819	52,348	37,498
Contigs without mt	36,809 (92.7%)	34,013 (94.2%)	22,983 (89%)	48,838 (93.3%)	34,592 (92.2%)
Singletons	184,139	171,709	217,290	263,428	168,075
Singletons without mt	65,066 (35.3%)	77,684 (45.2%)	56,705 (26.1%)	85,666 (32.5%)	69,968 (41.6%)
Total	223,862	207,797	243,109	315,776	205,573
Total without mt	101,875 (46%)	111,697 (54%)	79,688 (33%)	134,504 (43%)	104,560 (51%)
Av. contig length	375	415	424	407	360
Max contig length	4,255	7,513	5,317	6,063	4,841
Cumul. contig length	15.0 Mb	15.2 Mb	10.1 Mb	21.6 Mb	13.8 Mb
Cumul. total length	48.2 Mb	39.9 Mb	53.9 Mb	69.0 Mb	37.6 Mb
Average reads/contig	9.5	9.6	13.2	11.9	11.9
Greater than 500 b	7,080	7,796	5,206	10,709	6,081
Greater than 1 Kb	1,570	1,805	1,269	2,792	1,386
Av. sequencing depth	3.2	2.9	3.1	3.7	3.2

### Homology assignment and consensus building

Our strategy (of which a schematic representation is depicted in Figure 1), based on successive BLAST searches, followed by assembly against reference sequences, was performed with *'LANE runner'*, an in-house JAVA application (available at http://www.reptilian-transcriptomes.org) that provides a user-friendly interface for (i) BLAST-aligning multiple sequences against selected databases with different parameters (Figure 1a) and (ii) automatically building consensus sequences (Figure 1b and 1c). First, the contigs originating from the NGen assembly were aligned using BLAST, together with the singletons, against the known mitochondrial (mt) genome of each sequenced species, as well as a non-coding RNA database (Ensembl v56; http://www.ensembl.org) of seven reference species (*G. gallus*, *T. guttata*, *A. carolinensis*, *H. sapiens*, *M. musculus*, *X. tropicalis*, and *D. rerio*). A total of 6% to 11% of the contigs and 55% to 74% of the singletons were identified as mt sequences (Table 2) and were thus used for the reconstruction of each species organelle's transcriptome. Less than 100 sequences of each species were uniquely linked to known non-coding RNA (data not shown), probably because of the selection of poly-A transcripts during the library preparation and the small size of these non-coding RNA databases. Second, and for each of the five species investigated, the non-mtDNA sequences were sequentially aligned (Figure 1a), using BLAST, against the *'Ensembl Coding v56' *database (including the coding sequence and the 5' and 3' UTRs), the *'Unigene February 2010' *database, and the '*Ensembl Genomic v56*' database. In each case, BLAST searches were performed against the same seven reference species (*G. gallus*, *T. guttata*, *A. carolinensis*, *H. sapiens*, *M. musculus*, *X. tropicalis*, and *D. rerio*) and the results were restricted to hits longer than 50 bp. All query sequences with a BLAST hit against Ensembl Coding or Unigene were aligned against the corresponding reference sequences and used for generating consensus sequences. Finally, the contigs and singletons still with no hit (orphan sequences) were aligned (using BLAST) against the consensus sequences built at the previous steps for the other four species. When a single sequence hit a reference ('one-to-one'; Figure 1b), the sequence was simply named (for homology assignment) after the reference. When multiple sequences hit a reference ('one-to-many'; Figure 1b), their relative positions, with potential intervening gaps (see Additional file 1, Table S1), were identified using the reference (Figure 1b), but the latter was ignored for establishing the consensus itself among the query sequences (Figure 1c). The consensus was also named after the reference for homology assignment.

**Figure 2:**
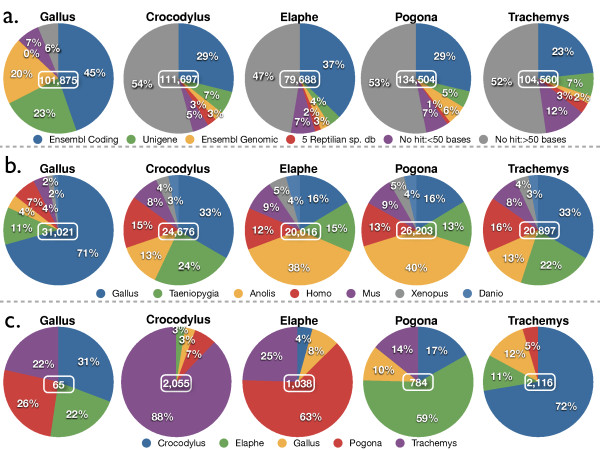
***'LANE Runner'*statistics**. **(a)** Percentages of contigs/singletons with a BLAST hit against each of the databases searched with '*LANE runner'*. The central number within each pie-chart is the number of contigs and singletons used in BLAST searches; **(b) **Percentage of each of the seven reference species used for anchoring input transcriptome sequences and building consensuses (results obtained against the Ensembl Coding and Unigene databases are grouped, and the central number gives the total number of consensuses); **(c) **Distribution of sequenced species against which 'orphan sequences' exhibited a hit (= fourth BLAST round in Figure 1a).

The left-most chart of Figure 2a indicates that the procedure works very efficiently: 87% of the *Gallus *input sequences can be attributed to a known sequence. This high success rate in homology assignment for chicken (*that is*, a reference species for which extensive whole-genome and transcriptome sequences are publicly available) strongly suggests that the lower percentage (about 50%) of hits observed for each of the other four species (Figure 2a) is simply due to (i) the lack of genome sequence data for the corresponding species and (ii) the large evolutionary distance between the species under study and the seven reference species used for BLAST. In other words, the chicken results indicate that the vast majority of the reptilian sequences reported here are very likely genuine transcripts. Note that the 50% of genes identified after our *de novo *sequencing of reptilian transcriptomes is larger than the proportion (about 25%) of transcript identification in previous efforts [27,51-53].

### Analyzing consensus sequences

Depending on the reptilian species, 36,241 to 54,284 input sequences (contigs + singletons) generated 20,016 to 26,203 consensus sequences (Table 3) of which the majority (>70%) were 'ono-to-one' consensuses. This result indicates that *de novo *sequencing of the brain transcriptome, as performed here, although it likely identifies the majority of the species' genes, does not provide large sequence coverage of each one. The lower percentage of 'one-to-one' consensuses obtained from the chicken brain transcriptome sequencing (88,754 input sequences generating 31,021 consensus sequences of which 58% are 'one-to-one'; Table 3) is due to the availability of the corresponding species genome/transcritome that facilitates the joining of contigs/singletons. This strongly suggests that some of the 'one-to-one' consensuses in the other species would be joined by additional sequencing efforts. Obviously, the efficiency of homology assignment decreases both with decreased query sequence length, and with increased evolutionary distance between the query sequences and the genomes/transcriptomes against which inter-species similarity (BLAST) searches are performed. For example, Figure S2 (Additional file 1) indicates that, for the species with no specific genome/transcriptome available, the mean size of input sequences (singletons and contigs) with a BLAST hit is substantially larger than that of sequences without a hit. It is likely that normalization of cDNA before sequencing and ongoing improvements in sequencing technology will help fill these gaps in transcriptome sequencing.

**Table 3:** **Consensus statistics**: number of sequences (total, contigs, and singletons) with a BLAST hit against reference databases, and number of consensus sequences generated

	*Gallus*	*Crocodylus*	*Elaphe*	*Pogona*	*Trachemys*
Input seq with BLAST hit	88,754	45,773	36,241	54,284	37,180
Contigs	35,330	18,505	13,530	24,981	17,534
Singletons	53,424	27,268	22,711	29,303	19,646
Consensus sequences	31,021	24,676	20,016	26,203	20,897
One-to-one consensus	17,885	19,617	15,701	18,802	17,728
>50% coverage	3,505	7,114	1,233	2,372	1,346

The sequencing of the reference species (chicken) highlights some important points. First, whereas only about half of the *Gallus *contigs and singletons exhibited a hit attributed to an Ensembl transcript (Figure 2a), almost a quarter matched a Unigene entry. Given that the latter database mainly consists of EST libraries, this result highlights the importance of splice variants and variable 3' UTRs. Furthermore, the 20% of the *Gallus *sequences that exhibited a BLAST hit against the chicken genome (and not against Ensembl Coding or Unigene databases) likely represent splice variants and novel transcript boundaries or unprocessed RNAs rather than genome contaminants, because 41% of them (8,173 reads out of 19,942) are found within gene boundaries, and an additional 35% are found in close proximity (within 5,000 bp of estimated boundaries). The fact that 87% of our *Gallus *transcriptome sequences could be identified is a clear indication that most of the reads represent existing expressed sequences, rather than sequencing or assembling artifacts. Note that more than half of the remaining 13% (*that is*, *Gallus *sequences with no hit) correspond to sequences that are less than 50 bp long and thus ignored during BLAST searches (Figure 2a), and/or are masked by the 'low complexity' filter applied (Additional file 1, Table S2).

Manual inspection of the resulting annotations indicates that, for each of the sequenced species, a substantial proportion of the longest contigs (as well as the contigs with the greatest number of reads) are homologized with transcripts known to be expressed in the brain (Additional file 1, Tables S3 and S4). For example, the myelin transcript was sequenced with one of the highest coverage for all species besides *Trachemys*. Other identified genes play a role in energy transduction or the organization of the cytoskeleton. The high complexity of the brain transcriptome also allows for the detection of transcripts with no known important function in the brain: *for example*, the testis-specific GAPDH-2 gene found in the *Elaphe *brain.

Most of the long contigs (and/or those with the largest number of reads) in *de novo *sequenced species do exhibit a BLAST hit. In fact, the rare cases of contigs >1,000 bp (and/or comprising >1,000 reads) that exhibit no BLAST hit against any of the reference species are very likely to represent species-specific transcripts and warrant additional investigation (Additional file 1, Table S5). Indeed, only a handful of the 966 species-specific transcripts give a significant BLAST hit against the 'nr' NCBI database, but they all (except 3 *Trachemys *sequences) exhibit a substantial ORF spanning, on average, 250 nucleotides.

### Phylogenetic distribution of BLAST hits

In Figure 2b, we show, for each sequenced species, the distribution of consensus sequences in seven categories, corresponding to the seven reference species against which consensus sequences were built. Whereas 71% of the *Gallus *consensus sequences were identified as *Gallus *transcripts, 11% generated a better hit with *Taeniopygia *sequences, suggesting that part of the zebra finch genome sequence might be of better quality than that of the chicken. In addition, 19% of the *Gallus *consensus sequences were assigned a best BLAST hit with yet a more distantly-related species. We assume that when the improved *Gallus *genome assembly [54] becomes available in Ensembl, an even greater percentage of best BLAST hits will be against the chicken genome. Because of the absence of reference genome/transcriptome sequences for the four other species, we expected the distribution of best BLAST hits to somewhat reflect the evolutionary distances between each sequenced species, on one hand, and each of the seven reference species, on the other hand. As anticipated (given the classical suggested grouping of crocodiles and birds into *Archosauria*), the majority of *Crocodylus *transcripts were assigned a best hit with a *Gallus *or *Taeniopygia *gene (33% and 24%, respectively). Similarly, *Anolis *is the species against which the largest number of *Elaphe *and *Pogona *transcripts exhibited a best BLAST hit, an expected result given the grouping of lizards and snakes into the Squamata clade [29]. More surprisingly, *Trachemys *exhibits the same pattern of BLAST-hits phylogenetic distribution as does *Crocodylus*, fueling the recent discussions on the possible close phylogenetic relationship between *Testudines *and *Archosauria *[55]. These evolutionary links are strikingly supported when the orphan sequences (*that is*, without a hit against any reference species) of each Sauropsida species are aligned (BLAST) against the built transcriptomes of the four others (Figure 2c): a majority of *Elaphe *orphan transcripts exhibit a best hit with *Pogona *transcripts (and *vice versa*), and a majority of *Crocodylus *orphan transcripts exhibit a best hit with *Trachemys *transcripts (and *vice versa*). Because similarity between molecular sequences is not only a function of the position of the corresponding lineages in the phylogeny, but also of the branch lengths of the gene tree, distances are imperfect indicators of phylogenetic relationships. We therefore performed extensive optimality-criterion-based phylogenetic analyses using massively-large multiple sequence alignments built from our transcriptomes data (see end of the Results section).

## Comparisons with existing transcriptome datasets

### Human and mouse databases

Because many of the consensus sequences corresponded to Ensembl transcripts, we identified the human or mouse homologs of these sequences (Table 4) using the Ensembl v56 BioMart database [56]. Recently, it was suggested that about 14,000 genes are expressed in the human and mouse brains [31]; we detect expression of an even greater number of genes in the brain of each of the sequenced reptilian species (Table 4). It was also suggested that 7,750 protein-coding genes are ubiquitously expressed across different human and mouse tissues and that the brain expresses one of the most complex transcriptomes [31]. We find that 4,124 to 5,822 (depending on the species) of these genes have a homolog in our sequenced transcriptomes, and that 2,595 of these genes are identified in all five species. Similarly, 6,922 to 9,020 of our sequenced genes are assigned as homologous to one of the 15,112 genes listed in the *'Mouse Brain Gene Atlas'*, an initiative aiming to identify, with cellular resolution, all genes expressed in the mouse brain [57].

**Table 4:** Comparisons with other transcriptome datasets: ubiquitously expressed genes [31] and the Mouse Brain Atlas [57]

	*Gallus*	*Crocodylus*	*Elaphe*	*Pogona*	*Trachemys*
Ensembl genes hits	17,346	18,407	17,335	20,964	15,101

Human homologs	10,425	8,167	8,658	9,964	6,940
	3,716 human homologs were found in all species				
7,750 ubiquitously expressed genes	5,822	4,804	5,097	5,752	4,124
	2,595 genes were found in all species				

Mouse homologs	11,238	10,068	10,162	11,697	8,970
	4,926 mouse homologs were found in all species				
15,112 Mouse Brain Atlas genes	8,873	7,844	7,907	9,020	6,922
	3,928 genes were found in all species				

### Avian and reptilian transcriptomes

The Ensembl *Gallus *genome contains about 18,000 genes, 9,515 of which we identified as partially sequenced for the chicken brain, confirming the large complexity of this organ's transcriptome. Similarly, when aligning (BLAST) all our chicken transcriptome sequences against the full chicken Unigene database or only the chicken brain Unigene database, we identified 61% or 82% of the known transcript clusters, respectively. These results show that a single deep-sequencing run can provide a large overview of a species' transcriptome in general, and a specific tissue transcriptome in particular. Similarly, despite the substantial divergence (about 150 million years of evolution) between *Pogona *and *Anolis*, we identified 8,397 of the approximately 21,000 *Anolis *Ensembl genes in the bearded dragon brain transcriptome. Given that the *Anolis *brain Unigene database mostly comprises EST libraries including a large proportion of fast-evolving 3' UTR sequences, only 25% of our *Pogona *sequences matched one of these Unigene clusters. In addition, among the 5,400 *A. missipiensis *ESTs available from a testis and liver library [24], 2,759 where hit by 2,000 of our 24,676 *Crocodylus *consensuses and by 2,395 of our 60,580 *Crocodylus *orphan sequences. This clearly demonstrates that (i) the sequences reported here correspond to genuine transcripts even if BLAST searches against the seven reference species did not identify homology, and (ii) joining contigs/singletons with consensus builds will require deeper transcriptome sequencing. Similarly, 1,252 of our *Trachemys *sequences BLAST-matched 587 (70%) of the 833 ESTs publicly available (NCBI) for that species. Again, note that half of these sequences were orphans when compared to the seven reference species.

### Thamnophis elegans transcriptome

The consensus and orphan sequences of the four reptilian species were compared to the *Thamnophis *recently-published transcriptome (containing 96,379 contigs and 92,561 singletons) [27]. As expected, *Elaphe *was the species with the greatest number of hits (15,121 consensus and 18,028 orphan sequences) thus reducing the percentage of orphan sequences from 47% to 34%. Although half of the *Pogona *consensus sequences had a match with the *Thamnophis *transcriptome, only 937 orphans had a hit. It is thus likely that the *Pogona *orphans represent fast-evolving sequences (like UTRs) and lineage-specific genes. The results for *Crocodylus *and *Trachemys *were similar to those of *Pogona *with, respectively, 0.12% and 0.2% of the orphan sequences matching the garter snake transcriptome.

### Gene Ontology Annotation

**Figure 3:**
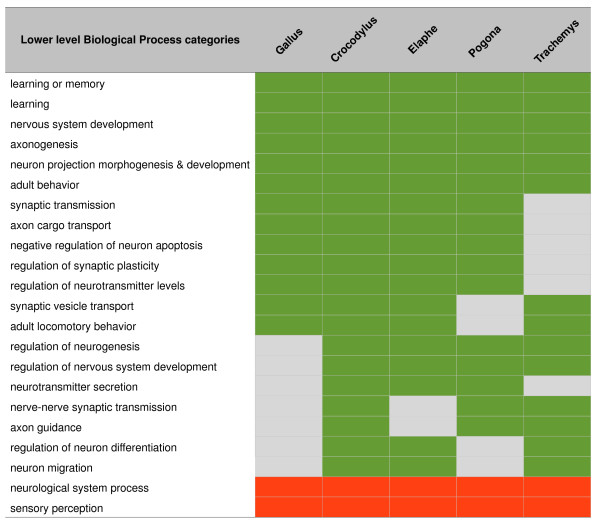
**Gene ontology low-level categories associated with central-nervous system biological processes for transcripts identified in each sequenced species**. Only the significantly over- and under-represented categories (False Discovery Rate <0.05) are shown, and they are marked with green and red, respectively (non-significant cases are in grey).

We investigated gene ontology annotation using GOSSIP [58] through the BLAST2GO platform [59]. The human homologs of the transcripts identified for each of the five Sauropsida species were compared to the full human gene set from Ensembl v56 for assessment of potential over- or under-representation of biological processes. Besides very obvious cases (such as the significant -- False Discovery Rate <0.05 -- under-representation in our brain transcriptomes of genes from the 'immune system' and 'reproductive processes' categories), the over- or under-representation of first-level categories are generally difficult to interpret because they comprise very diverse lower-level categories, and each gene may belong to more than one category. On the other hand, many of the lower-level categories associated with central-nervous system functions are over-represented in the brain transcriptome of one or more of the species of interest (Figure 3). Note that the significant under-representation of 'neurological system processes' and 'sensory perception' genes in the brain transcriptome of all five species is not as surprising as it might seem. Indeed, these categories group genes that are directly expressed in sensory organs rather than in the central nervous system.

### Identification of SSR loci and SNPs

It was long assumed that simple sequence repeats (SSRs or microsatellites) were primarily associated with non-coding DNA, but it has now become clear that they are frequently located in transcribed sequences. SSRs can affect gene silencing and transcription, as well as mRNA splicing, export, and translation, such that they probably impact on organism development, adaptation, survival, and evolution [60]. Screening of all our contigs and singletons yielded, per sequenced species, 7,378 to 15,986 SSRs made of 1 to 6 base-long repeats (*that is*, 0.07 to 0.12 microsatellite per sequence; Additional file 1, Table S6). For all species, mononucleotides exceed all other repeat types, except for *Elaphe *and *Pogona *that exhibited a similar number of mono- and dinucleotide repeats. Frequencies of SSR categories decrease with increasing repeat size, in contradiction with the suggested higher number of trinucleotide than dinucleotide repeats in coding sequences [61]; but our dataset also includes 3' and 5' UTRs which are not constrained by codons. A/T and AC/GT are the most abundant mono- and dinucleotide repeats, respectively, except for *Elaphe *where AG/CT is the dominant dinucleotide. The prominent trinucleotide in *Crocodylus *is AAT/ATT, and AGG/CCT for the remaining four species. The most frequent tetra-, penta- and hexanucleotides are (AT)-rich, as reported for non-mammalian vertebrates [61,62].

By screening all our contigs, we initially identified 39,907 to 122,790 SNPs per sequenced species. However, given the risk of false positives (due to sequencing errors), we constructed a high-confidence SNP set by selecting only those with a minimum minor allele frequency of three (thus a minimum sequencing depth of six). The number of high-confidence SNPs varied from 1,808 in *Crocodylus *to 18,710 in *Pogona*, and the greatest number (4.87) per contig was observed in *Elaphe *(Additional file 1, Table S7). Similarly, we initially identified 51,719 to 127,926 insertion/deletion polymorphisms (indels) per sequenced species. Selecting only indel sites with a minimum minor allele frequency of 3 reduces these numbers to 11,916 - 36,276. Note however that 14.3% to 18.8% of these were included in homopolymers, hence, are low confidence indel sites because pyrosequencing tends to generate insertion/deletions errors when encountering homopolymers [63]. The high-confidence SNPs and Indels are all potential valuable markers for linkage analyses.

### Mitochondrial genomes

The mitochondrial sequences were assembled against and compared with a reference mtDNA genome sequence (NCBI) for each species. In all cases, our assembly covered all mt genes except for 1 to 14 tRNAs (depending on the species) and part of the control region (Additional file 1, Table S8). Surprisingly, a large portion of the NADH dehydrogenase subunit 5 gene was lacking in our *Crocodylus *and *Pogona *transcriptome sequences. As expected, transitions outnumbered transversions in the comparisons with the reference conspecific sequence, and very few of the coding-sequence mutations occurred at the second codon position (suggesting that all these substitutions are genuine mtDNA SNPs).

### Initial phylogeny inference

Reptilian phylogeny has been heavily debated, especially regarding the placement of the *Testudines *(turtles) clade. Indeed, given that they lack temporal skull openings (Anapsida condition), turtles have traditionally been considered as the basal linage of amniotes [64,65], *that is*, the sister group to those reptiles with two temporal skull openings (Diapsida condition): Archosauria (crocodiles and birds) and Lepidosauria (tuatara, lizards, and snakes). This view has recently been challenged by morphological analyses (*for example*, [66], but see [67,68]) suggesting that turtles may be diapsids that secondarily lost skull temporal fenestration. Similarly, several molecular phylogenetic studies either suggested a sister relationship of turtles with a monophyletic Archosauria (crocodiles and birds) [69-73], or joined crocodilians with turtles to the exclusion of birds [74-76]. Potential affinity of turtles and crocodilians has also been observed when evaluating the history of amniote genomes and genomic signatures [35].

Here, we used our transcriptome sequence data for performing maximum likelihood (ML) molecular phylogenetic analyses of very large multiple-sequence alignments to identify the placement of turtles within the phylogeny of amniotes. The advantages of our approach are twofold: the number of characters is exceptionally large and character sampling is widely distributed across the genome, reducing the risk of gene-specific biases potentially present in previous analyses. The disadvantages of our dataset are its low species sampling and the high error rates generated by low-depth sequencing.

**Figure 4:**
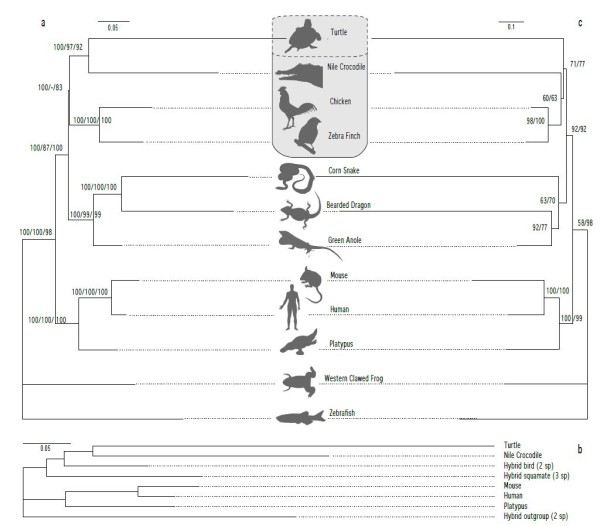
**Phylogenomic analyses**. **(a)** Amino-acid sequences from 4,689 genes (for 7 Sauropsida, 3 mammals, and 2 outgroup taxa) analyzed with *RaxML *(WAG model and approximate rate heterogeneity) after removal of excessively gapped positions (final dataset size = 2,012,759 characters/species), as well as with *RaxML *(GTR model and approximate rate heterogeneity) and *MetaPIGA-2 *(GTR, gamma-rate heterogeneity) after removal of all gapped positions (final size of dataset = 1,612 characters per species); labels on nodes indicate bootstrap proportions under *RaxML *for the 2 million and 1,612 aa datasets as well as posterior probabilities generated by *MetaPIGA *for the 1,612 aa dataset; branch lengths are indicated for the *MetaPIGA *analysis. **(b)** Amino-acid sequences from 4,689 genes (for 5 Sauropsida lineages, including 2 hybrid sequences, 3 mammals, and 1 hybrid outgroup) after removal of all gapped positions (final dataset size = 24,071 characters/species) analyzed with *RaxML *(GTR, approximate rate heterogeneity) and *MetaPIGA *(GTR, gamma-rate heterogeneity); bootstrap proportions and posterior probabilities are 100% for all branches; *MetaPIGA *branch lengths are indicated. **(c) **Amino-acid sequences from 1,139 genes devoid of known paralogs (for 7 Sauropsida species, 3 mammals and 2 outgroup taxa) analyzed with *MetaPIGA *after removal of excessively gapped positions (final size of dataset = 246,208 characters/species); labels on nodes indicate posterior probabilities for analyses under GTR with/without gamma rate heterogeneity; analysis of this dataset under *RaxML* still generated long-branch attraction: (corn snake + bearded dragon) and (crocodile + turtle).

For each of the 4,689 proteins identified in both the crocodile and turtle transcriptomes, we performed a multiple alignment with the homologous sequences (when available) from each of our 3 other transcriptomes (corn snake, bearded dragon, and chicken) as well as from 8 species in Ensembl *v56*: Anole lizard, zebra finch, Western clawed frog, zebra fish, chicken, mouse, human, and Platypus. This generated a 12-species multiple alignment with >3.3 million aa residues per species. Note however that for many of the (incompletely) sequenced proteins, residue positions can be lacking for one or more of the 12 taxa, generating a mosaic dataset with different species lacking different genes or portions of them. Incomplete datasets must be analyzed with caution because recent simulation studies indicate that missing data can seriously affect the accuracy of phylogenetic estimates [77]. One effective solution is the removal of positions on the basis of both gap and similarity distributions in the alignment, *that is*, favoring the removal of positions with most gaps and most divergent residues. Using such an automated procedure (*trimAl *[46]), we generated a 12-species alignment with 2,012,759 aa columns that we analyzed under ML (with WAG substitution model) using RaxML [48]. This analysis generated a highly-supported tree topology with turtles as the sister group to crocodiles (Figure 4a). More drastically, we removed all columns including at least one gap, *that is*, keeping only the characters represented in all 12 species, and generated an alignment of only 1,612 columns. Analyses of this dataset using *RaxML* or the meta-population Genetic Algorithm [50] with *MetaPIGA-2 *[49] under the General-Time-Reversible (GTR) substitution model (with or without rate heterogeneity) all yield the same sister-group relationship between crocodile and turtle (Figure 4a). Note however that the RaxML analysis grouped birds with Lepidosauria instead of Archosauria. One solution for keeping both a large number of characters and a small number of missing data is to combine (hybridize) sequences (Additional file 1, Figure S4) from species that clearly form monophyletic groups. For example, combining the two birds (*Gallus *and *Taeniopygia*), combining the two non-amniote outgroup species (*Danio *and *Xenopus*), and combining the three squamates (anole lizard, bearded dragon, and corn snake), before removing gaps from the alignment, generates a gap-free 8-terminal taxa dataset with 24,071 aligned aa rather than a 12-species dataset with 1,612 columns. *RaxML* and *MetaPIGA* analyses (GTR with rate heterogeneity) of this hybrid dataset again strongly group birds as the sister group to [crocodile + turtle] (Figure 4b). Changing the species priority within hybrid sequences (*for example*, favoring *Gallus *to *Taeniopygia*) did not change the resulting topology.

### Phylogeny inference after removing paralogs

The meta-analyses performed above can still suffer from a major problem: it is highly likely that some of the sequences included in our multi-species multi-gene alignments are paralogous, hence generating potential artifacts (possibly with high support values) during phylogeny inference. In an attempt to exclude that problem, we used *MANTiS*[47] to select, in our reptilian transcriptome dataset, the protein sequences for which the corresponding gene in *Danio*, *Xenopus*, *Anolis*, *Gallus*, *Taeniopygia*, *Mus*, *Homo*, and *Platypus *exhibits no known paralog. Given that these eight genomes are of high quality, it is likely that all paralogs have been identified such that our approach drastically reduces the risk of comparing paralogs in the final alignment. Trimming the selected aligned sequences using gap and similarity distributions yielded a final alignment of 246,208 columns × 12 species. *MetaPIGA* analyses (GTR model with or without rate heterogeneity) of this very large dataset yield the tree in Figure 4c: remarkably, the grouping of snake and bearded dragon (to the exclusion of the anole lizard) observed in the previous analyses (Figure 4a) disappears and is replaced by the traditional grouping of the agamid and iguanid lizard families to the exclusion of snakes, suggesting that indeed paralogous sequences generated an artifact in that portion of the tree. Similarly, these *MetaPIGA* analyses excluding paralogs do not group anymore turtles and crocodiles to the exclusion of birds, but position turtles as the sister-group to a monophyletic Archosauria (crocodiles + birds). Note that the analysis of the same dataset under *RaxML* (GTR and rate heterogeneity) still generated, in our hands, long-branch attractions (corn snake + bearded dragon as well as crocodile + turtle), possibly because of the rate-heterogeneity approximations used by this program.

## Conclusions

We report here on the first large-scale comparative analysis of transcriptomes across Sauropsida, a group clearly more speciose than mammals. Such transcriptome resources will become increasingly important as reptiles are emerging models for developmental [37,78,79], as well as ecological and physiological, studies [4,80-84].

We show that our automated pipeline (*LANErunner*) performing (i) recursive BLAST searches against both Ensembl Coding (including genes predicted on the basis of evidence from closely-related species) and Unigene (including ESTs) databases from reference species covering a wide range of evolutionary distances, and (ii) consensus building, is highly efficient compared to less flexible available scripts. For example, the recent analysis of a garter snake multi-individual and multi-organ transcriptome [27] identified 25% of the assembled contigs and singletons, whereas we identified >50% of our corn snake non-redundant sequences. In addition, the extensive use of the Ensembl database gives easy access to other types of information, such as gene ontology. Our dataset also provides an extensive Sauropsida resource for microsatellite (SSRs) and polymorphic markers (despite the fact that we started from a single individual brain for each species) for designing population genetics or linkage studies. Finally, we used our transcriptome data for generating very large sequence alignments and performing extensive maximum likelihood phylogenetic analyses (under the GTR model of aa substitution with rate heterogeneity) to elucidate the highly controversial phylogenetic position of turtles.

Our data and analyses are not without limitations. First, the use of brain tissue for transcriptome sequencing reduces, but does not necessarily eliminate, the need for normalization. Most importantly, removal of mitochondrial transcripts is warranted, either through normalization or removal of these organelles. In addition, despite the fact that the brain exhibits one of the most complex transcriptomes, dynamic range of mRNA expression might be high enough to warrant normalization before *de novo *sequencing. Second, identifying the presence of genes and transcripts does not equate with building a phylome, *that is*, the complete set of gene family trees across species (as performed in Ensembl [85] or the PhylomeDB [86]). Phylomes are of great interest in evolutionary biology because they allow rigorous differentiation of orthologs and paralogs, hence, the identification of gene gains, duplications, and losses on the species phylogenetic tree [47,87], as well as the investigation of gene expression evolution [88-90]. However, data from low-depth sequencing (as used here) can generate gene-tree building errors that, in turn, can cause striking artifacts in gene duplication inference, especially at the most recent common ancestor of low-coverage genomes/transcriptomes [91]. Regarding species phylogeny inference, undetected sequencing errors (probably partially responsible for the long terminal branches of the corresponding taxa; Figure 4) might also have generated artifactual grouping of long-branches [92] as illustrated, in some analyses, by the incorrect grouping of the bearded dragon lizard with the corn snake to the exclusion of the green anole lizard (Figure 4a). In addition, given that we did not generate a phylome, it is likely that some of the sequences included in our initial multi-species multi-gene alignments were paralogous, hence, generating additional potential artifacts during phylogeny inference. In this respect, it is remarkable that we could recover the correct grouping of the bearded dragon lizard with the green anole lizard to the exclusion of the corn snake, when performing ML analyses (using a GTR model of aa substitution with or without rate-heterogeneity in *MetaPIGA-2*; [49]) of a large alignment of protein-coding genes with no known paralogs in high-quality full genomes (Figure 4c). These results suggest that the sister-group relationship of turtles with a monophyletic Archosauria (birds + crocodiles) uncovered under these settings (Figure 4c) constitutes the best-supported phylogenetic position of turtles, making them highly-derived diapsid reptiles which secondarily lost their skull temporal fenestration.

Until additional deep-sequencing is performed, the data and analyses reported here provide, for the major lineages of Sauropsida (crocodiles, snakes and lizards, turtles, and birds), identification of a majority of transcripts (*for example*, for future gene expression analyses), detection of thousands of microsatellites, thousands of SNP and indel polymorphisms (*for example*, for future quantitative genetics and population genetic analyses), as well as yield the largest phylogenomic dataset to date for the investigation of a long-standing question: the phylogenetic position of turtles in the vertebrate evolutionary tree. All developed software tools and sequence data generated by these analyses are freely available at http://www.reptilian-transcriptomes.org.

## Competing interests

The authors declare that they have no competing interests.

## Authors' contributions

MCM and ACT conceived the study. ACT performed tissue processing and cDNA library construction. GS performed sequencing and initial quality control of reads. ACT, MCM, and RH conceived the automated pipeline '*LANErunner*' which was then coded by RH. ACT performed contig initial assembly, homology assignments, building and analysis of consensus sequences, and identification of SSR loci and SNPs. ACT and MCM performed the phylogenetic analyses. ACT built the mySQL database and the PHP query web interface. MCM made the http://www.reptilian-transcriptomes.org web site. MCM, ACT, and RH wrote the manuscript. All authors read and approved the final manuscript.

## Authors' information

MCM heads the Laboratory of Artificial & Natural Evolution (LANE) in the Department of Genetics and Evolution at the University of Geneva (Switzerland), and works on various aspects of evolutionary developmental genetics, phylogenomics, phyloinformatics, and conservation genetics. ACT is a post-doctoral fellow at the LANE and works on comparative transcriptomics and evolutionary developmental genetics in mammals and reptiles. RH is a computer scientist in the de Duve Institute (Belgium) and specializes in the development of bioinformatic tools. GS is a computer scientist at Roche.

## Supplementary Material

Additional file 1 **additional technical figures and tables**. Contigs, BLAST search, gaps, and microsatellites/SNPs statistics, hybrid sequence principle. Click here for file
