# Supplementary material for: Reptilian-transcriptome v1.0, a glimpse in the brain transcriptome of five divergent Sauropsida lineages and the phylogenetic position of turtles
Source: EvoDevo. 2011 Sep 26;2:19. doi: 10.1186/2041-9139-2-19 (PMC3192992; doi:10.1186/2041-9139-2-19)
Supplement: Additional file 1 — additional technical figures and tables. Contigs, BLAST search, gaps, and microsatellites/SNPs statistics, hybrid sequence principle. [file 2041-9139-2-19-S1.PDF]

Additional file 1.

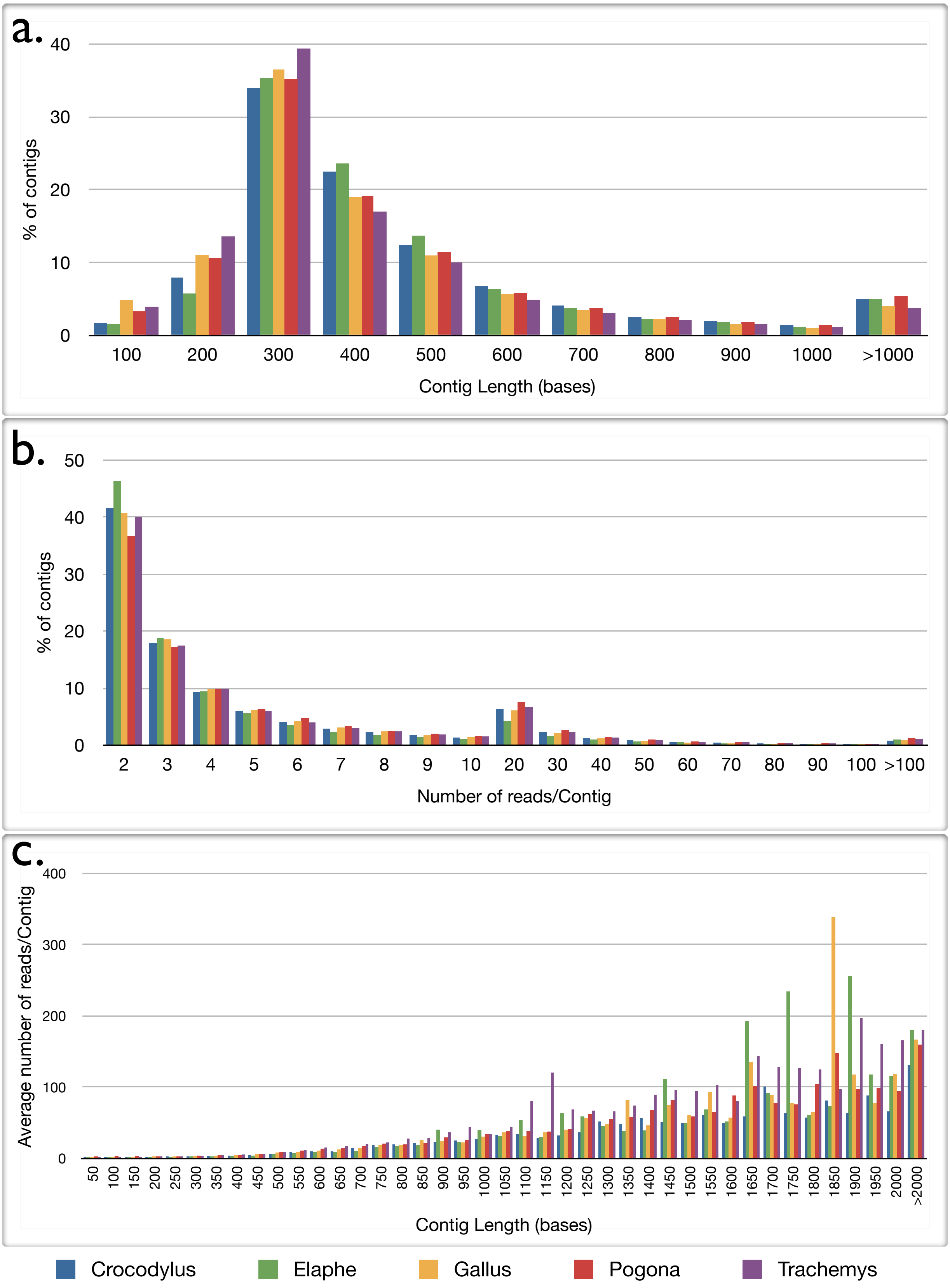

Figure S1. Contig statistics (a) Distribution of contig lengths, (b) Distribution of number of reads/contig, and (c) Average number of reads per contig *versus* contig length.

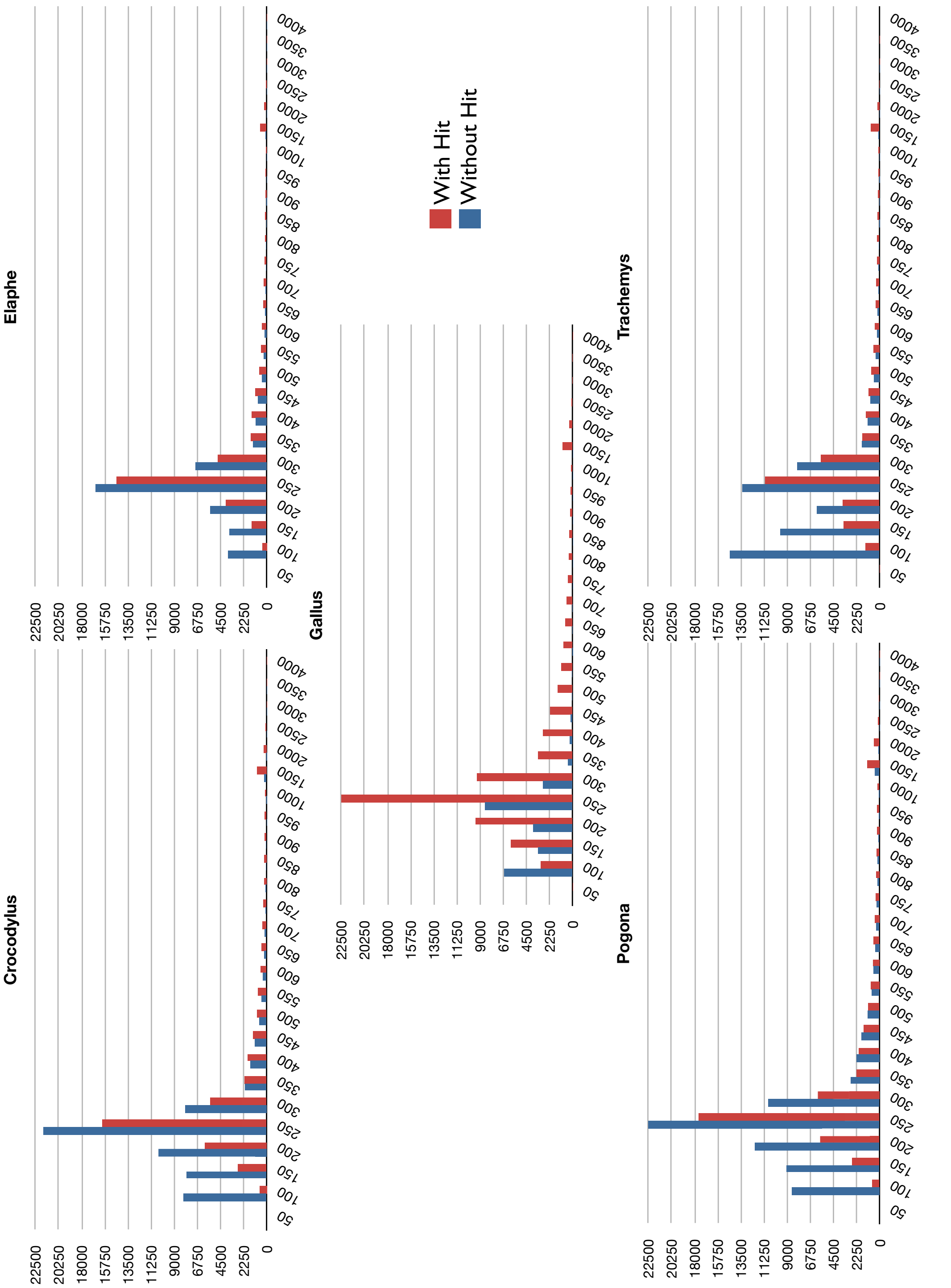

Figure S2. Size distribution of the contigs and singletons with and without a BLAST hit.

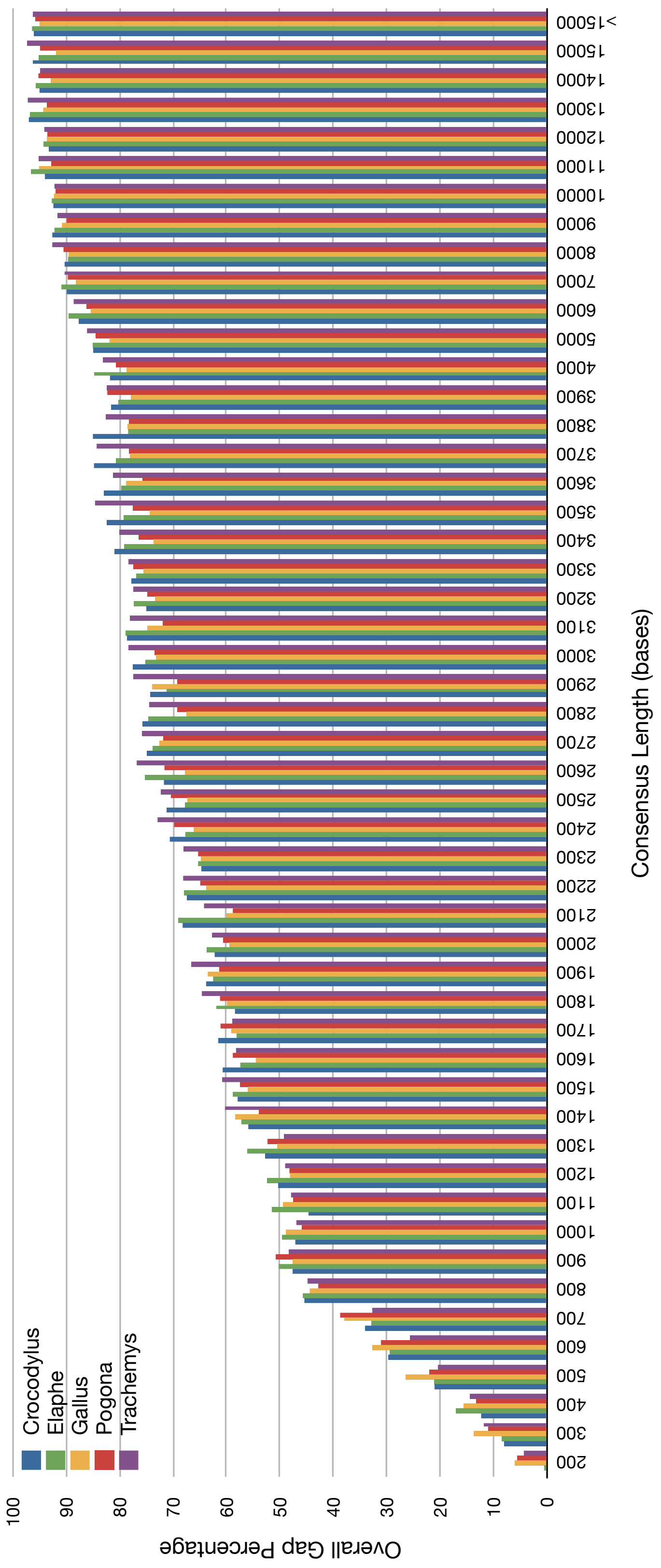

Figure S3. Gap percentage *versus* consensus sequences length.

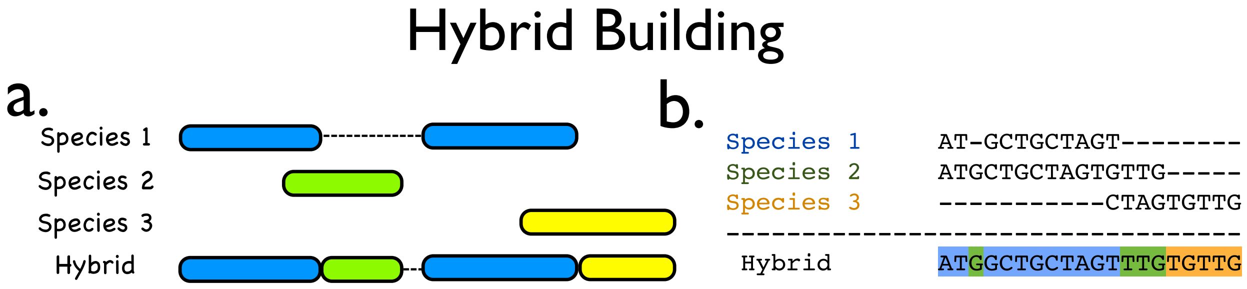

Figure S4. Description of how hybrid sequences are built (here, with species 1 prioritized) for the large-scale phylogenetic analyses.

Table S1. 'One-to-many' consensus sequences and gap percentages. Note that our library are 3'-enriched given that polyT primers were used during RNA amplification for a better estimate of the number of unique transcripts [1].

	Gallus	Crocodylus	Elaphe	Pogona	Trachemys
Consensus sequences	31,086	26,731	21,054	26,987	23,013
'One-to-many' consensuses	13,201	7,114	5,353	8,185	5,285
3'-end gap percentage	69.6	70.4	65.6	67.5	70.8
Middle gap percentage	61.7	63.1	62	58.7	62.5
5'-end gap percentage	55.6	59.7	65.6	58.2	58.4
Overall gap percentage	62.2	64.3	64.1	61.2	63.8
<10% gaps	919	567	400	758	469
Consensus longer than reference	10,525	6,057	4,358	6,772	4,588
Average number of additional bases	68	79.5	83	111	83.5

Table S2. RepeatMasker results for the input sequences with no BLAST hit.

	Gallus	Crocodylus	Elaphe	Pogona	Trachemys
Number of input sequences without BLAST hit	6,620	61,132	39,453	71,973	55,531
Masked sequences	848	5,686	5,883	7,747	6,065
Sequences with < 50 bases after masking	385	552	698	603	925

Table S3. Annotation of the ten longest contigs per species (the contigs with known expression in the brain are marked in grey).

Name	Reference Transcript	Reference Description	Length	# of reads	Reference
Croc_00282	ENSGALT00000026187	ATPase, H+ transporting, lysosomal accessory protein 2 [NP_001025972]	3878	152	
Croc_04316	ENSGALT00000011913	Not Available	3709	86	
Croc_00156	ENST00000296755	Microtubule-associated protein 1B (MAP-1B) [Contains MAP1 light chain LC1] [P46821]	3522	216	[21]
Croc_00601	ENSGALT00000000305	Dihydropyrimidinase-related protein 2 (DRP-2) (Collapsin response mediator protein CRMP-62) [Q90635]	3506	197	[2]
Croc_00501	Genomic DNA	Not Available	3417	237	
Croc_00509	ENSACAT00000010422	Synaptic vesicle membrane protein VAT-1 homolog-like [Q9HCJ6]	3299	183	[20]
Croc_04219	ENSGALT00000006719	hexokinase 1 [NP_989432]	3286	106	
Croc_00685	ENSGALT00000023996	Gap junction alpha-1 protein (Connexin-43)(Cx43) [P14154]	3273	97	

Croc_00287	Gga.34997	Gallus gallus finished cDNA, clone ChEST79b1	3160	248	www.ncbi.nlm.nih.gov/nucest/25346210
Croc_00985	ENSGALT00000035062	Calsyntenin-1 protein Fragment [Q9DDD3]	3158	126	
Elaphe_00406	ENSACAT00000017046	2',3'-cyclic-nucleotide 3'-phosphodiesterase (CNPase)(CNP)(EC 3.1.4.37) [P09543]	4910	218	[3]
Elaphe_00470	ENST00000358196	Glutamate decarboxylase 1 (EC 4.1.1.15)(Glutamate decarboxylase 67 kDa isoform)(67 kDa glutamic acid decarboxylase)(GAD-67) [Q99259]	4576	129	[19]
Elaphe_00712	ENSTGUT00000004332	Synaptic vesicle glycoprotein 2C [Taeniopygia guttata]	4302	148	[4]
Elaphe_00927	ENSXETT00000026418	Amyloid-like protein 1 Precursor (APLP-1)(APLP) [Contains C30] [P51693]	4054	336	[18]
Elaphe_00435	ENSGALT00000001057	Not Available	3916	80	
Elaphe_09213	ENSGALT00000026733	Not Available	3870	91	
Elaphe_00384	Genomic DNA	Not Available	3842	150	
Elaphe_00105	ENSACAT00000001420	Serine incorporator 1 (Tumor differentially expressed protein 2)(Tumor differentially expressed 1 protein-like) [Q9NRX5]	3743	316	
Elaphe_00478	ENSGALT00000036985	V-type proton ATPase catalytic subunit A (V-ATPase subunit A)(EC 3.6.3.14)(Vacuolar proton pump subunit alpha)(V-ATPase 69 kDa subunit) [Q90647]	3691	160	
Elaphe_00276	ENSXETT00000016776	Calcium/calmodulin-dependent protein kinase type 1B (EC 2.7.11.17)(CaM kinase I beta)(CaM kinase IB)(CaM-KI beta)(CaMKI-beta)(Pregnancy up-regulated non-ubiquitously-expressed CaM kinase) [Q6P2M8]	3617	236	
Gallus_02681	ENSGALT00000005668	Na+-dependent glutamate/aspartate transporter Fragment [Q9IAS3]	4255	149	
Gallus_01226	ENSGALT00000040758	hexokinase 1 [NP_989432]	3925	192	
Gallus_00563	ENSGALT00000039574	T-cell immunomodulatory protein [NP_001005840]	3905	150	
Gallus_00196	ENST00000398238	Vesicle-fusing ATPase (EC 3.6.4.6)(N-ethylmaleimide-sensitive fusion protein)(NEM-sensitive fusion protein)(Vesicular-fusion protein NSF) [P46459]	3757	217	
Gallus_00530	ENSGALT00000031997	reticulon 4 [NP_989697]	3568	291	[5]
Gallus_01214	ENSGALT00000039859	Eukaryotic translation initiation factor 4 gamma 2 Fragment (eIF-4-gamma 2)(eIF-4G 2)(eIF4G 2)(p97) [O73777]	3358	179	
Gallus_00326	ENSGALT00000026753	glycoprotein M6B [NP_001012563]	3343	186	[17]
Gallus_00548	ENSGALT00000015643	Protein NEL Precursor (93 kDa protein) [Q90827]	3302	275	[6]
Gallus_00072	ENSGALT00000039314	glucose phosphate isomerase [NP_001006128]	3237	257	
Gallus_00309	ENSGALT00000019661	Ankyrin 2 Fragment [Q90716]	3197	215	
Pogona_01276	ENST00000398238	Vesicle-fusing ATPase (EC 3.6.4.6)(N-ethylmaleimide-sensitive fusion protein)(NEM-sensitive fusion protein)(Vesicular-fusion protein NSF) [P46459]	6063	264	
Pogona_01058	ENST00000326427	Integral membrane protein 2C (Transmembrane protein BRI3)(Cerebral protein 14) [Contains CT-BRI3] [Q9NQX7]	4788	295	[16]
Pogona_00437	ENSGALT00000040351	calnexin [NP_001025791]	4689	274	
Pogona_01600	ENSACAT00000012153	Myristoylated alanine-rich C-kinase substrate (MARCKS)(Protein kinase C substrate, 80 kDa protein, light chain)(PKCSL)(80K-L protein) [P29966]	4520	279	
Pogona_01757	ENSACAT00000001420	Serine incorporator 1 (Tumor differentially expressed protein 2)(Tumor differentially expressed 1 protein-like) [Q9NRX5]	4486	242	
Pogona_04310	Genomic DNA	Not Available	4217	202	
Pogona_00912	Acr.4302	Acr#S49084302 G1144P348FA24.T0 Anolis carolinensis pooled normalized embryo cDNA library Anolis carolinensis cDNA, mRNA sequence /gb=FG719868 / gi=190249086	4063	253	
Pogona_01888	ENSACAT00000000247	Not Available	4047	230	
Pogona_00823	ENSGALT00000034651	Not Available	4019	239	

Pogona_03066	ENST00000273398	V-type proton ATPase catalytic subunit A (V-ATPase subunit A)(EC 3.6.3.14)(Vacuolar proton pump subunit alpha)(V-ATPase 69 kDa subunit)(Vacuolar ATPase isoform VA68) [P38606]	3896	177	
Trachemys_00149	ENSGALT00000038920	Profilin [Q5ZL50]	3223	170	
Trachemys_00278	ENSGALT00000041230	matrin 3 [NP_989478]	3001	199	
Trachemys_00807	ENST00000264710	Ras-related protein Rab-10 [P61026]	2942	101	[7]
Trachemys_00511	ENSTGUT00000004278	Major prion protein Precursor (PrP)(PrP27-30)(PrP33-35C)(ASCR)(CD230 antigen) [Taeniopygia guttata]	2845	183	www.ncbi.nlm.nih.gov/protein/123303164
Trachemys_01162	ENSGALT00000027597	ubiquitin-like 3 [NP_001012847]	2788	155	
Trachemys_00629	Hs.621695	gnl|UG|Hs#S36661991 Homo sapiens nuclear enriched abundant transcript 2 (NEAT2) mRNA, complete sequence /gb=EF177381 /gi=124294889	2738	215	
Trachemys_00429	ENSTGUT00000000416	Thy-1 membrane glycoprotein Precursor (Thy-1 antigen)(CDw90)(CD90 antigen) [Taeniopygia guttata]	2737	154	[15]
Trachemys_00040	ENSDART00000028338	Secretory carrier-associated membrane protein 5 (Secretory carrier membrane protein 5) [Q6P0C7]	2694	143	
Trachemys_00755	ENSACAT00000011808	14-3-3 protein beta/alpha (Protein kinase C inhibitor protein 1)(KCIP-1)(Protein 1054) [Contains 14-3-3 protein beta/alpha, N-terminally processed] [P31946]	2693	145	
Trachemys_00342	ENSDART00000011878	eukaryotic translation initiation factor 4A, isoform 1B [NP_958918]	2683	193	

Table S4. Annotation of the ten contigs with the greatest number of reads per species (the contigs with known expression in the brain are marked in grey).

Name	Reference Transcript	Reference Description	Length	# of reads	Reference
Croc_Repeat-31467	ENSGALT00000022185	Myelin basic protein (MBP) [P15720]	2356	1766	[8]
Croc_Repeat-31464	ENSACAT00000000543	Actin, aortic smooth muscle (Alpha-actin-2)(Cell growth-inhibiting gene 46 protein) [P62736]	2145	1524	
Croc_Repeat-31466	ENST00000361621	Myelin proteolipid protein (PLP)(Lipophilin) [P60201]	2459	1169	[14]
Croc_Repeat-31471	ENST00000254976	Synaptosomal-associated protein 25 (SNAP-25)(Super protein)(SUP) [P60880]	2102	787	[9]
Croc_Repeat-31477	ENSACAT00000015718	Tubulin alpha-1A chain (Tubulin B-alpha-1)(Tubulin alpha-3 chain)(Alpha-tubulin 3) [Q71U36]	1688	778	[13]
Croc_Repeat-31470	ENST00000381694	Creatine kinase, ubiquitous mitochondrial Precursor (EC 2.7.3.2)(U-MtCK)(Acidic-type mitochondrial creatine kinase)(Mia-CK) [P12532]	1674	642	www.uniprot.org/uniprot/P12532
Croc_Repeat-31482	Tgu.4295	gnl|UG|Tgu#S33940999 Taeniopygia guttata clone 0058P0018F02 calcium/calmodulin-dependent protein kinase II variant 2-like mRNA, complete sequence /gb=DQ216257 /gi=76159676	1222	603	www.ncbi.nlm.nih.gov/nuccore/76159676
Croc_Repeat-31517	ENSTGUT00000000923	Cold-inducible RNA-binding protein (Glycine-rich RNA-binding protein CIRP)(A18 hnRNP) [Taeniopygia guttata]	1293	599	
Croc_Repeat-31483	ENSTGUT00000000329	Ornithine decarboxylase antizyme (ODC-Az) [Taeniopygia guttata]	1030	590	
Croc_Repeat-31472	ENSGALT00000037622	Neo-calmodulin Fragment (NeoCaM) [P05419]	1395	550	
Elaphe_Repeat-21098	ENSGALT00000016997	Tubulin alpha-1 chain Fragment [P02552]	1712	3668	
Elaphe_Repeat-21088	ENSTGUT00000009443	Myelin basic protein (MBP)(Myelin A1 protein)(Myelin membrane encephalitogenic protein) [Taeniopygia guttata]	2493	3503	[8]

Elaphe_Repeat-21096	ENSACAT00000010124	Glyceraldehyde-3-phosphate dehydrogenase, testis-specific (EC 1.2.1.12)(Spermatogenic glyceraldehyde-3-phosphate dehydrogenase)(Spermatogenic cell-specific glyceraldehyde 3-phosphate dehydrogenase 2)(GAPDH-2) [O14556]	1638	3110	
Elaphe_Repeat-21091	ENSMUST00000031564	Actin, cytoplasmic 1 (Beta-actin) [Contains Actin, cytoplasmic 1, N-terminally processed] [P60710]	2002	2609	
Elaphe_Repeat-21090	PogonaNH_323624_2290_1355	Not available	1432	2604	
Elaphe_Repeat-21095	ENSTGUT00000013012	Calmodulin (CaM) [Taeniopygia guttata]	1893	2428	
Elaphe_Repeat-21093	ENSGALT00000041222	Ornithine decarboxylase antizyme (ODC-Az) [O42148]	1197	2414	
Elaphe_Repeat-21099	ENSACAT00000017183	L-lactate dehydrogenase B chain (LDH-B)(EC 1.1.1.27)(LDH heart subunit)(LDH-H)(Renal carcinoma antigen NY-REN-46) [P07195]	2389	2325	
Elaphe_Repeat-21109	ENSXETT00000045926	Hypothetical protein MGC75752. [Q6P4N0]	862	2150	
Elaphe_Repeat-21119	ENSXETT00000052176	Not available	987	1947	
Gallus_Repeat-33956	ENSGALT00000029104	Not available	1824	3961	
Gallus_Repeat-33960	ENSDART00000067321	Microtubule-associated protein 1A (MAP-1A)(Proliferation-related protein p80) [Contains MAP1 light chain LC2] [P78559]	2053	3083	
Gallus_Repeat-33957	ENSGALT00000022185	Myelin basic protein (MBP) [P15720]	2669	2666	[8]
Gallus_Repeat-33963	ENSXETT00000017485	Not available	1633	2042	
Gallus_Repeat-33961	ENSGALT00000037122	Glyceraldehyde-3-phosphate dehydrogenase (GAPDH)(EC 1.2.1.12) [P00356]	1316	2026	
Gallus_Repeat-33958	ENSGALT00000038297	Calmodulin (CaM) [P62149]	1836	1699	
Gallus_Repeat-33998	ENSTGUT00000013337	Not available	2952	1376	
Gallus_Repeat-33962	ENSGALT00000019594	reticulon 1 [NP_001001466]	1518	1182	[10]
Gallus_Repeat-34067	ENSGALT00000040933	Heat shock cognate 71 kDa protein (Heat shock 70 kDa protein 8) [O73885]	2326	1030	
Gallus_Repeat-33967	ENST00000423183	Not available	1865	987	
Pogona_Repeat-44802	ENSTGUT00000009443	Myelin basic protein (MBP)(Myelin A1 protein)(Myelin membrane encephalitogenic protein) [Taeniopygia guttata]	2393	4068	[8]
Pogona_Repeat-44801	No BLAST hit	Not available	1814	3838	
Pogona_Repeat-44930	ENSDART00000093609	Cytochrome c oxidase subunit 2 (EC 1.9.3.1)(Cytochrome c oxidase polypeptide II) [Q9MIY7]	566	2329	
Pogona_Repeat-44805	ENST00000320936	Cold-inducible RNA-binding protein (Glycine-rich RNA-binding protein CIRP)(A18 hnRNP) [Q14011]	1442	1968	
Pogona_Repeat-44817	ENSMUST00000077577	Tubulin alpha-1B chain (Tubulin alpha-2 chain)(Alpha-tubulin 2)(Alpha-tubulin isotype M-alpha-2) [P05213]	1769	1959	[11]
Pogona_Repeat-44821	ENST00000221476	Creatine kinase M-type (EC 2.7.3.2)(Creatine kinase M chain)(M-CK) [P06732]	1558	1824	www.uniprot.org/uniprot/P06732
Pogona_Repeat-44818	ENSMUST00000004673	Protein NDRG2 (Protein Ndr2) [Q9QYG0]	3041	1761	[12]
Pogona_Repeat-44811	ENSTGUT00000001168	UPF0471 protein C1orf63 [Taeniopygia guttata]	2433	1693	
Pogona_Repeat-44804	ENSACAT00000004488	Not available	1228	1555	
Pogona_Repeat-44851	Acr.874	gnl|UG|Acr#S49014010 G1142P32RB15.T0 Anolis carolinensis pooled normalized Brain cDNA Library Anolis carolinensis cDNA, mRNA sequence / gb=FG649576 /gi=190156236	1607	1294	www.ncbi.nlm.nih.gov/nucest/190156236
Trachemys_Repeat-32622	ENSGALT00000029106	Not available	1114	6896	
Trachemys_Repeat-32229	ENSXETT00000008566	Not available	2158	3381	
Trachemys_Repeat-32224	ENSGALT00000041222	Ornithine decarboxylase antizyme (ODC-Az) [SO42148]	1078	2029	
Trachemys_Repeat-32239	ENSMUST00000058914	Tubulin alpha-1C chain (Tubulin alpha-6 chain)(Alpha-tubulin 6)(Alpha-tubulin isotype M-alpha-6) [P68373]	1645	2010	

Trachemys_Repeat-32232	ENSXETT00000057558	Actin, cytoplasmic, type 5. [Q6P378]	1892	1942	
Trachemys_Repeat-32225	ENSGALT00000037622	Neo-calmodulin Fragment (NeoCaM) [P05419]	1690	1574	
Trachemys_Repeat-32248	ENSACAT00000001904	Synaptosomal-associated protein 25 (SNAP-25) (Synaptosomal-associated 25 kDa protein)(Super protein)(SUP) [P60880]	1950	1096	[9]
Trachemys_Repeat-32289	ENSGALT00000014913	Not available	1762	987	
Trachemys_Repeat-32266	ENSACAT00000000769	Creatine kinase B-type (EC 2.7.3.2)(Creatine kinase B chain)(B-CK) [P12277]	1436	986	www.uniprot.org/uniprot/P12277
Trachemys_Repeat-32254	ENSACAT00000004488	Not available	1196	923	

Table S5. Number of Contigs longer than 1,000 bases or comprising more than 1,000 reads.

Contigs	Gallus	Crocodylus	Elaphe	Pogona	Trachemys	All
Total number	39,723	36,088	25,819	52,348	37,498	191,476
>1,000 bp	1,575	1,808	1,270	2,798	1,392	8,843
>1,000 bp with BLAST hit	1,574	1,545	1,145	2,366	1,247	7,877
Possibly species-specific	1	263	125	432	145	966
with >1,000 reads	20	11	28	29	14	102
with >1,000 reads and a BLAST hit	20	11	28	27	14	100

Table S6. Microsatellite statistics. The minimum number of repeats is indicated between parentheses. A 'compound' microsatellite corresponds to two or more microsatellites separated by less than 50 bp.

	Gallus	Crocodylus	Elaphe	Pogona	Trachemys	All (sum or average)
Number of microsats	7,378	11,451	9,694	15,986	12,993	57,502
Average microsat/input sequence	0.07	0.10	0.12	0.12	0.12	0.11
Mononucleotides (>10)	4,264	7,620	3,049	6,397	9,362	30,692
Dinucleotides (>5)	2,066	3,057	3,838	6,345	3,064	18,370
Trinucleotides (>5)	818	547	1,785	2,272	422	5,844
Tetranucleotides (>5)	139	136	766	900	110	2051
Pentanucleotides (>5)	69	74	229	63	29	464
Hexanucleotides (>5)	22	17	27	9	6	81
Compounds	782	1,196	1,289	1,861	1,315	6,443

Table S7. Single nucleotide and indel polymorphism statistics (mitochondrial DNA sequences are excluded); *ma*= minimum allele; *Av*= average.

	Gallus	Crocodylus	Elaphe	Pogona	Trachemys	All
Number of SNPs	46,878	57,316	39,907	122,790	77,559	344,450
SNPs with ma depth>3 (SNP3)	3,091	1,808	3,905	18,710	8,044	35,558
Av. nr SNPs/contig with SNP3	2.8	2.9	4.9	4.5	3.8	3.8
Number of Indels	55,429	77,107	51,719	127,926	107,445	419,626
Indels with ma depth>3	12,129	16,882	11,916	36,276	35,338	112,541
Indels (ma depth>3) within mononucl. repeats (≥4)	1,906	3,173	2,115	5,301	5,037	17,532
High-quality indels	10,223	13,709	9,801	30,975	30,301	95,009
Av. Indels/contig	3.6	4.6	6.1	5.2	6.9	5.3

Table S8. mtDNA analyses.

	Gallus	Crocodylus	Elaphe	Pogona	Trachemys
Transitions	30	127	11	71	19
Transversions	2	10	2	24	0
Gaps	2,790	1,875	674	868	213
Modified amino acids	5	16	1	19	5
2nd position mutations	4	4	0	8	2
Missing tRNAs	14	6	2	2	1

Bibliography

1. Emrich SJ, Barbazuk WB, Li L, Schnable PS: **Gene discovery and annotation using LCM-454 transcriptome sequencing.** *Genome Res* 2007, **17:**69-73.
2. Goshima Y, Nakamura F, Strittmatter P, Strittmatter SM: **Collapsin-induced growth cone collapse mediated by an intracellular protein related to UNC-33.** *Nature* 1995, **376:**509-514.
3. Sakamoto Y, Tanaka N, Ichimiya T, Kurihara T, Nakamura KT: **Crystal structure of the catalytic fragment of human brain 2',3'-cyclic-nucleotide 3'-phosphodiesterase.** *J Mol Biol* 2005, **346:**789-800.
4. Gingrich JA, Andersen PH, Tiberi M, el Mestikawy S, Jorgensen PN, Fremeau RT, Jr., Caron MG: **Identification, characterization, and molecular cloning of a novel transporter-like protein localized to the central nervous system.** *FEBS Lett* 1992, **312:**115-122.
5. Caltharp SA, Pira CU, Mishima N, Youngdale EN, McNeill DS, Liwnicz BH, Oberg KC: **NOGO-A induction and localization during chick brain development indicate a role disparate from neurite outgrowth inhibition.** *BMC Dev Biol* 2007, **7:**32.
6. Matsuhashi S, Noji S, Koyama E, Myokai F, Ohuchi H, Taniguchi S, Hori K: **New gene, nel, encoding a M(r) 93 K protein with EGF-like repeats is strongly expressed in neural tissues of early stage chick embryos.** *Dev Dyn* 1995, **203:**212-222.
7. Hu RM, Han ZG, Song HD, Peng YD, Huang QH, Ren SX, Gu YJ, Huang CH, Li YB, Jiang CL, Fu G, Zhang QH, Gu BW, Dai M, Mao YF, Gao GF, Rong R, Ye M, Zhou J, Xu SH, Gu J, Shi JX, Jin WR, Zhang CK, Wu TM, Huang GY, Chen Z, Chen MD, Chen JL: **Gene expression profiling in the human hypothalamus-pituitary-adrenal axis and full-length cDNA cloning.** *Proc Natl Acad Sci U S A* 2000, **97:**9543-9548.
8. Li H, Richardson WD: **The evolution of Olig genes and their roles in myelination.** *Neuron Glia Biol* 2008, **4:**129-135.
9. Bark IC, Wilson MC: **Human cDNA clones encoding two different isoforms of the nerve terminal protein SNAP-25.** *Gene* 1994, **139:**291-292.
10. Baka ID, Ninkina NN, Pinon LG, Adu J, Davies AM, Georgiev GP, Buchman VL: **Intracellular compartmentalization of two differentially spliced s-rex/NSP mRNAs in neurons.** *Mol Cell Neurosci* 1996, **7:**289-303.
11. Lewis SA, Lee MG, Cowan NJ: **Five mouse tubulin isotypes and their regulated expression during development.** *J Cell Biol* 1985, **101:**852-861.
12. Hu XL, Liu XP, Deng YC, Lin SX, Wu L, Zhang J, Wang LF, Wang XB, Li X, Shen L, Zhang YQ, Yao LB: **Expression analysis of the NDRG2 gene in mouse embryonic and adult tissues.** *Cell Tissue Res* 2006, **325:**67-76.
13. Poirier K, Keays DA, Francis F, Saillour Y, Bahi N, Manouvrier S, Fallet-Bianco C, Pasquier L, Toutain A, Tuy FP, Bienvenu T, Joriot S, Odent S, Ville D, Desguerre I, Goldenberg A, Moutard ML, Fryns JP, van Esch H, Harvey RJ, Siebold C, Flint J, Beldjord C, Chelly J: **Large spectrum of lissencephaly and pachygyria phenotypes resulting from de novo missense mutations in tubulin alpha 1A (TUBA1A).** *Hum Mutat* 2007, **28:**1055-1064.
14. Popot JL, Pham Dinh D, Dautigny A: **Major myelin proteolipid: the 4-alpha-helix topology.** *J Membr Biol* 1991, **123:**278.
15. Porrero C, Rubio-Garrido P, Avendano C, Clasca F: **Mapping of fluorescent protein-expressing neurons and axon pathways in adult and developing Thy1-eYFP-H transgenic mice.** *Brain Res* 2010, **1345:**59-72.

16. Wickham L, Benjannet S, Marcinkiewicz E, Chretien M, Seidah NG: **Beta-amyloid protein converting enzyme 1 and brain-specific type II membrane protein BRI3: binding partners processed by furin.** *J Neurochem* 2005, **92:**93-102.
17. Schweitzer J, Becker T, Schachner M, Nave KA, Werner H: **Evolution of myelin proteolipid proteins: gene duplication in teleosts and expression pattern divergence.** *Mol Cell Neurosci* 2006, **31:**161-177.
18. Kim TW, Wu K, Xu JL, McAuliffe G, Tanzi RE, Wasco W, Black IB: **Selective localization of amyloid precursor-like protein 1 in the cerebral cortex postsynaptic density.** *Brain Res Mol Brain Res* 1995, **32:**36-44.
19. Chessler SD, Lernmark A: **Alternative splicing of GAD67 results in the synthesis of a third form of glutamic-acid decarboxylase in human islets and other non-neural tissues.** *J Biol Chem* 2000, **275:**5188-5192.
20. Nagase T, Kikuno R, Hattori A, Kondo Y, Okumura K, Ohara O: **Prediction of the coding sequences of unidentified human genes. XIX. The complete sequences of 100 new cDNA clones from brain which code for large proteins in vitro.** *DNA Res* 2000, **7:**347-355.
21. Ding J, Liu JJ, Kowal AS, Nardine T, Bhattacharya P, Lee A, Yang Y: **Microtubule-associated protein 1B: a neuronal binding partner for gigaxonin.** *J Cell Biol* 2002, **158:**427-433.
